# Sex Differences in Prehospital Identification of Large Vessel Occlusion in Patients With Suspected Stroke

**DOI:** 10.1161/STROKEAHA.123.044898

**Published:** 2024-02-01

**Authors:** Mariam Ali, Luuk Dekker, Jasper D. Daems, Mahsoem Ali, Erik W. van Zwet, Ewout W. Steyerberg, Martijne H.C. Duvekot, T. Truc My Nguyen, Walid Moudrous, Ruben M. van de Wijdeven, Marieke C. Visser, Karlijn F. de Laat, Henk Kerkhoff, Ido R. van den Wijngaard, Diederik W.J. Dippel, Bob Roozenbeek, Nyika D. Kruyt, Marieke J.H. Wermer

**Affiliations:** 1 Department of Neurology (Mariam Ali, L.D., I.R.v.d.W., N.D.K., M.J.H.W., T.T.M.N.), Leiden University Medical Center, the Netherlands.; 2 Department of Biomedical Data Sciences (E.W.v.Z., E.W.S.), Leiden University Medical Center, the Netherlands.; 3 Department of Neurology (M.C.V.); 4 Department of Surgery (Mahsoem Ali); 5 Amsterdam UMC, Vrije Universiteit Amsterdam, the Netherlands (M.C.V.).; 6 Department of Neurology (J.D.D., M.H.C.D., R.M.v.d.W., D.W.J.D., B.R.), Erasmus MC University Medical Center, Rotterdam, the Netherlands.; 7 Department of Public Health (J.D.D.), Erasmus MC University Medical Center, Rotterdam, the Netherlands.; 8 Department of Neurology, Albert Schweitzer Hospital, Dordrecht, the Netherlands (M.H.C.D., H.K.).; 9 Department of Neurology, Maasstad Hospital, Rotterdam, the Netherlands (W.M.).; 10 Department of Neurology, Haga Hospital, the Hague, the Netherlands (K.F.d.L.).; 11 Department of Neurology, Haaglanden Medical Center, the Hague, the Netherlands (I.R.v.d.W.).; 12 University Neurovascular Center Leiden-The Hague, the Netherlands (I.R.v.d.W., N.D.K.).; 13 Department of Neurology, University Medical Centre Groningen, University of Groningen, the Netherlands (M.J.H.W.).

**Keywords:** acute ischemic stroke, emergency medical services, large vessel occlusion, sex, thrombectomy

## Abstract

**BACKGROUND:**

Differences in clinical presentation of acute ischemic stroke between men and women may affect prehospital identification of anterior circulation large vessel occlusion (aLVO). We assessed sex differences in diagnostic performance of 8 prehospital scales to detect aLVO.

**METHODS:**

We analyzed pooled individual patient data from 2 prospective cohort studies (LPSS [Leiden Prehospital Stroke Study] and PRESTO [Prehospital Triage of Patients With Suspected Stroke Study]) conducted in the Netherlands between 2018 and 2019, including consecutive patients ≥18 years suspected of acute stroke who presented within 6 hours after symptom onset. Ambulance paramedics assessed clinical items from 8 prehospital aLVO detection scales: Los Angeles Motor Scale, Rapid Arterial Occlusion Evaluation, Cincinnati Stroke Triage Assessment Tool, Cincinnati Prehospital Stroke Scale, Prehospital Acute Stroke Severity, gaze-face-arm-speech-time, Conveniently Grasped Field Assessment Stroke Triage, and Face-Arm-Speech-Time Plus Severe Arm or Leg Motor Deficit. We assessed the diagnostic performance of these scales for identifying aLVO at prespecified cut points for men and women.

**RESULTS:**

Of 2358 patients with suspected stroke (median age, 73 years; 47% women), 231 (10%) had aLVO (100/1114 [9%] women and 131/1244 [11%] men). The area under the curve of the scales ranged from 0.70 (95% CI, 0.65–0.75) to 0.77 (95% CI, 0.73–0.82) in women versus 0.69 (95% CI, 0.64–0.73) to 0.75 (95% CI, 0.71–0.79) in men. Positive predictive values ranged from 0.23 (95% CI, 0.20–0.27) to 0.29 (95% CI, 0.26–0.31) in women versus 0.29 (95% CI, 0.24–0.33) to 0.37 (95% CI, 0.32–0.43) in men. Negative predictive values were similar (0.95 [95% CI, 0.94–0.96] to 0.98 [95% CI, 0.97–0.98] in women versus 0.94 [95% CI, 0.93–0.95] to 0.96 [95% CI, 0.94–0.97] in men). Sensitivity of the scales was slightly higher in women than in men (0.53 [95% CI, 0.43–0.63] to 0.76 [95% CI, 0.68–0.84] versus 0.49 [95% CI, 0.40–0.57] to 0.63 [95% CI, 0.55–0.73]), whereas specificity was lower (0.79 [95% CI, 0.76–0.81] to 0.87 [95% CI, 0.84–0.89] versus 0.82 [95% CI, 0.79–0.84] to 0.90 [95% CI, 0.88–0.91]). Rapid arterial occlusion evaluation showed the highest positive predictive values in both sexes (0.29 in women and 0.37 in men), reflecting the different event rates.

**CONCLUSIONS:**

aLVO scales show similar diagnostic performance in both sexes. The rapid arterial occlusion evaluation scale may help optimize prehospital transport decision-making in men as well as in women with suspected stroke.

The clinical benefit of endovascular thrombectomy (EVT) for patients with acute ischemic stroke with anterior circulation large vessel occlusion (aLVO) is strongly time-dependent.^1,2^ Therefore, several prehospital scales have been developed to identify patients with aLVO enabling direct transportation to an EVT-capable stroke center, with subsequent reduced time delays in the initiation of EVT.

**See related article, p 555**

It is unknown whether differences according to sex affect the diagnostic performance of these aLVO scales. In a recent meta-analysis on sex differences in clinical presentation of acute stroke, we found that focal stroke symptoms, including hemiparesis, are less common in women than in men, whereas the opposite holds for nonfocal symptoms.^3^ Stroke presentation with nonfocal symptoms is associated with a higher risk of misdiagnosis.^4^ Interestingly, a previous cohort study found that women under 70 with stroke were more likely to be diagnosed by paramedics with conditions other than stroke.^5^ In addition, the higher frequency of stroke mimics in women could also affect diagnostic performance of these scales.^6^

Therefore, we aimed to evaluate the sex-specific diagnostic performance of 8 commonly used aLVO scales.^7–14^

## METHODS

### Data Availability Statement

In compliance with Dutch law, patient data cannot be made available, since participants were not informed during the opt-out procedure about the public sharing of their individual participant data in deidentified form. The syntax and output files of the statistical analyses can be made available from the corresponding author upon reasonable request.

### Study Design and Study Population

This study followed the STARD (Standards for Reporting of Diagnostic Accuracy Studies) reporting guideline for diagnostic accuracy studies (Supplemental Material).^15^ We used pooled data from the LPSS (Leiden Prehospital Stroke Study) and the PRESTO study (Prehospital Triage of Patients With Suspected Stroke).^16,17^ Both were prospective, multicenter, observational cohort studies, which included patients aged 18 years and older with suspicion of acute stroke as assessed by ambulance paramedics and who presented within 6 hours after symptom onset. Ambulance paramedics conducted assessments for all patients with suspected stroke in the prehospital setting, examining clinical items of prehospital aLVO detection scales. In PRESTO, we assessed the following 9 items: facial palsy, arm motor function, leg motor function, abnormal speech, gaze deviation, agnosia, grip strength, answering questions, and following commands. The LPSS study included these and added sensory deficits and tactile extinction assessments. An overview of the included clinical items in each scale is provided in Table S1. Stroke suspicion was defined by a positive Face-Arm-Speech-Time test (PRESTO) or any other deficits suspected for stroke (LPSS). Data were obtained from 15 hospitals in 4 ambulance regions in the southwest of the Netherlands from 2018 to 2019.

### Data

Available data in the pooled LPSS/PRESTO database included sex, age, baseline blood pressure, prehospital stroke scale assessment by paramedics, stroke severity according to the National Institutes of Health Stroke Scale at the emergency department, scores on the individual National Institutes of Health Stroke Scale items, medical history, medication use, prestroke modified Rankin Scale score, and center type (primary stroke center versus comprehensive stroke center).^18,19^

### aLVO Scales

Ambulance paramedics used a mobile application to score clinical items on scene or during transport before hospital arrival, which enabled reconstruction of 8 aLVO scales: Los Angeles Motor Scale,^7^ Rapid Arterial Occlusion Evaluation (RACE),^8^ Cincinnati Stroke Triage Assessment Tool,^9^ Cincinnati Prehospital Stroke Scale,^10^ Prehospital Acute Stroke Severity,^11^ gaze-face-arm-speech-time,^12^ Conveniently Grasped Field Assessment Stroke Triage,^13^ and face-arm-speech-time plus severe arm or leg motor deficit.^14^

All scales had a prespecified cut point to detect aLVO, except for the face-arm-speech-time plus severe arm or leg motor deficit test, which required a positive FAST-test and severe paresis of at least 1 limb to generate a positive face-arm-speech-time plus severe arm or leg motor deficit test score. For the Cincinnati Prehospital Stroke Scale, we used a modified cut point of ≥3, as this cut point has previously been suggested for LVO detection.^20^

### Outcome

The main outcome was a clinical diagnosis of ischemic stroke combined with aLVO on computed tomography angiography (intracranial carotid artery, tandem intracranial carotid artery, M1/M2 segments of the middle cerebral artery, and A1/A2 segments of the anterior cerebral artery) based on local assessment (LPSS) or assessment by an Imaging Core Laboratory (PRESTO). Diagnostic performance of the 8 aLVO scales was assessed separately for men and women in terms of area under the curve, sensitivity, specificity, positive predictive value (PPV), and negative predictive value using the cut points defined in the original studies, except for the modified cut point for the Cincinnati Prehospital Stroke Scale.

### Statistical Analysis

We reported baseline characteristics of men and women as number (%), mean (SD), or median (interquartile range) as appropriate. A 2-sided *P*<0.05 was considered statistically significant for baseline characteristics.

Diagnostic performance of aLVO scales was assessed for men and women separately, with corresponding 95% CIs by computing 10 000 stratified bootstrap replicates. The χ^2^ test was used to compare performance measures between sexes.

A 2-sided *P*<0.05 was considered statistically significant with a Benjamini-Hochberg multiple test correction to control the study wise false discovery rate for each performance measure separately^.21^

Multiple imputation was performed for missing clinical items of aLVO scales in the LPSS study using the mice package generating 5 imputation datasets with plausible values for these missing items. Each data set was created through an imputation model that estimated the missing values based on other observed variables. We then calculated performance metrics (area under the curve, sensitivity, specificity, PPV, and negative predictive value) for each aLVO scale separately across all 5 imputed datasets. These individual results were pooled into a single summary using Rubin Rules by calculating the mean values and mean 95% CIs of these measures across the datasets. In PRESTO, there were no missing items. Statistical analyses were performed using R version 4.1.2 (R Foundation for Statistical Computing).

Details of missing data per scale are provided in Table S2, while Table S3 shows missing scores for each prehospital aLVO scale stratified by sex. Diagnostic performance of scales, stratified by cohort, is in Tables S4 and S5, and for the subgroup of patients with confirmed stroke in Table S6, including ischemic stroke, intracranial hemorrhage, and transient ischemic attack.

### Ethical Statement

The LPSS and PRESTO studies were reviewed by the relevant medical ethical review committees and approved by the institutional review boards of the participating centers. The need for informed consent was waived. More detailed information about these methods is described elsewhere.^16,17^

## RESULTS

Of 2358 patients with suspected stroke, 1114 (47%) were women, and median (IQR) age was 73 (61–81) years (Figure). Women less often had a medical history of hyperlipidemia (52% versus 57%; *P*=0.005), myocardial infarction (6% versus 15%; *P*<0.001), and peripheral arterial disease (3% versus 5%; *P*=0.02) than men (Table 1). In addition, the use of antiplatelet therapy was less prevalent in women (34% versus 39%; *P*=0.004), whereas a moderate to severe prestroke disability (modified Rankin Scale score, 3–5) was more prevalent in women than in men (17% versus 12%; *P*<0.001).

**Table 1.**
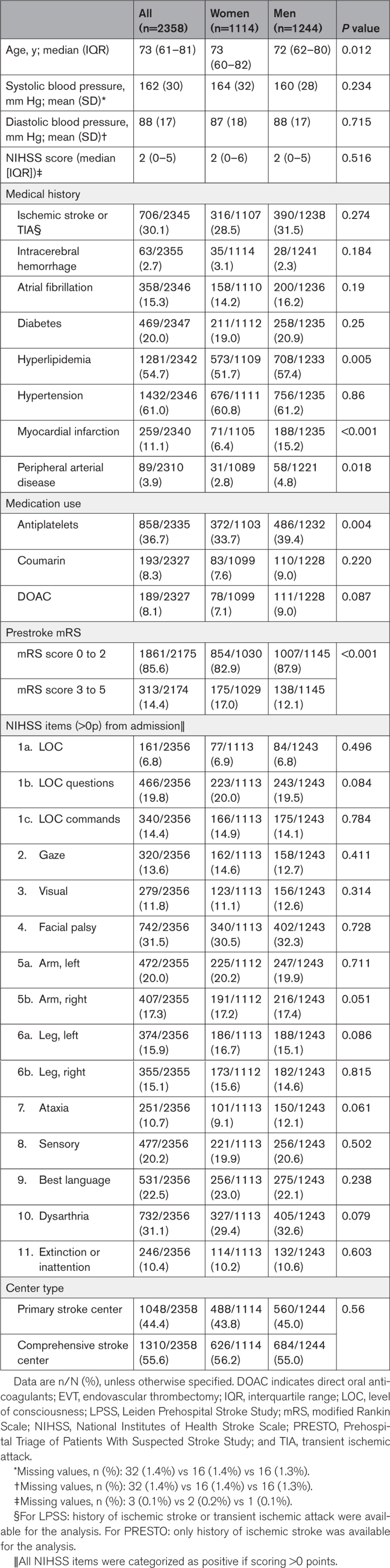
Baseline Characteristics of the Participants Stratified by Sex

**Figure.**
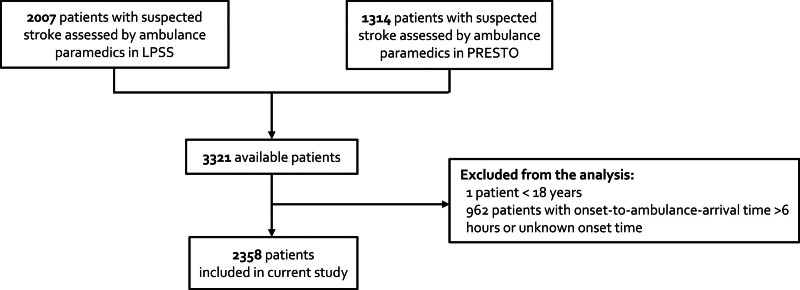
Flow diagram of patients in the LPSS (Leiden Prehospital Stroke Study) and PRESTO study (Prehospital Triage of Patients With Suspected Stroke).

The median National Institutes of Health Stroke Scale score at admission was similar in men and women (median, 2 [IQR, 0–6] in women versus 2 [IQR, 0–5] in men; *P*=0.52). Women had a slightly lower frequency of dysarthria (29% versus 33%; *P*=0.08). Almost the same proportion of women and men first presented in a primary stroke center (44% versus 45%; *P*=0.56).

### Outcome

Overall, 231 patients received a diagnosis of ischemic stroke with aLVO. The prevalence of aLVO was somewhat lower among women (100/1114 [9%]) than men (131/1244 [11%]; Table 2) but similar among women (100/460 [22%]) and men (131/602 [22%]) diagnosed with ischemic stroke. There were substantial differences in diagnosis between men and women. Stroke mimics were diagnosed more frequently in women (36% versus 26%; *P*<0.0001), whereas non-aLVO ischemic stroke was diagnosed less frequently in women than in men (32% versus 38%; *P*=0.005).

**Table 2.**
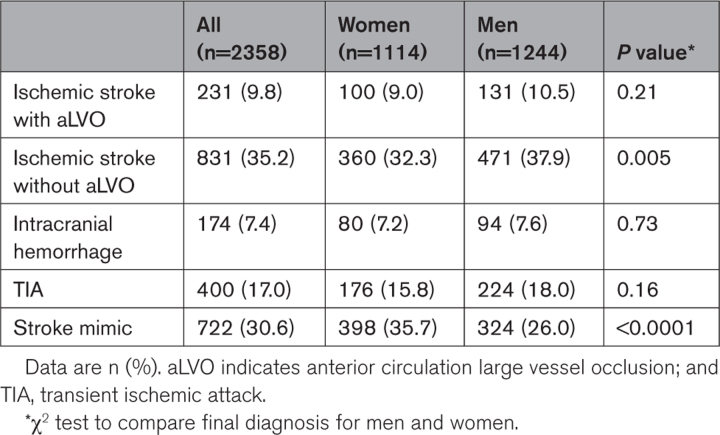
Final Diagnosis Stratified by Sex

### Scale Performance

The discriminative abilities of the individual scales ranged from an area under the curve of 0.70 (95% CI, 0.65–0.75) for the Conveniently Grasped Field Assessment Stroke Triage scale to 0.77 (95% CI, 0.73–0.82) for the Los Angeles Motor Scale in women. In men, the AUC ranged from 0.69 (95% CI, 0.64–0.73) for the Cincinnati Stroke Triage Assessment Tool scale to 0.75 (95% CI, 0.71–0.79) for the RACE scale (Table 3). PPV of the scales ranged from 0.23 (95% CI, 0.20–0.27) to 0.29 (95% CI, 0.26–0.31) in women versus 0.29 (95% CI, 0.24–0.33) to 0.37 (95% CI, 0.32–0.43) in men, with RACE having the highest PPV in both sexes (0.29 in women and 0.37 in men). Negative predictive value were similar (0.95 [95% CI, 0.94–0.96] to 0.98 [95% CI, 0.97–0.98] in women versus 0.94 [95% CI, 0.93–0.95] to 0.96 [95% CI, 0.94–0.97] in men). Sensitivity of the scales was slightly higher in women than in men (0.53 [95% CI, 0.43–0.63] to 0.76 [95% CI, 0.68–0.84] versus 0.49 [95% CI, 0.40–0.57] to 0.63 [95% CI, 0.55–0.73]), whereas specificity was lower (0.79 [95% CI, 0.76–0.81] to 0.87 [95% CI, 0.84–0.89] versus 0.82 [95% CI, 0.79–0.84] to 0.90 [95% CI, 0.88–0.91]).

**Table 3.**
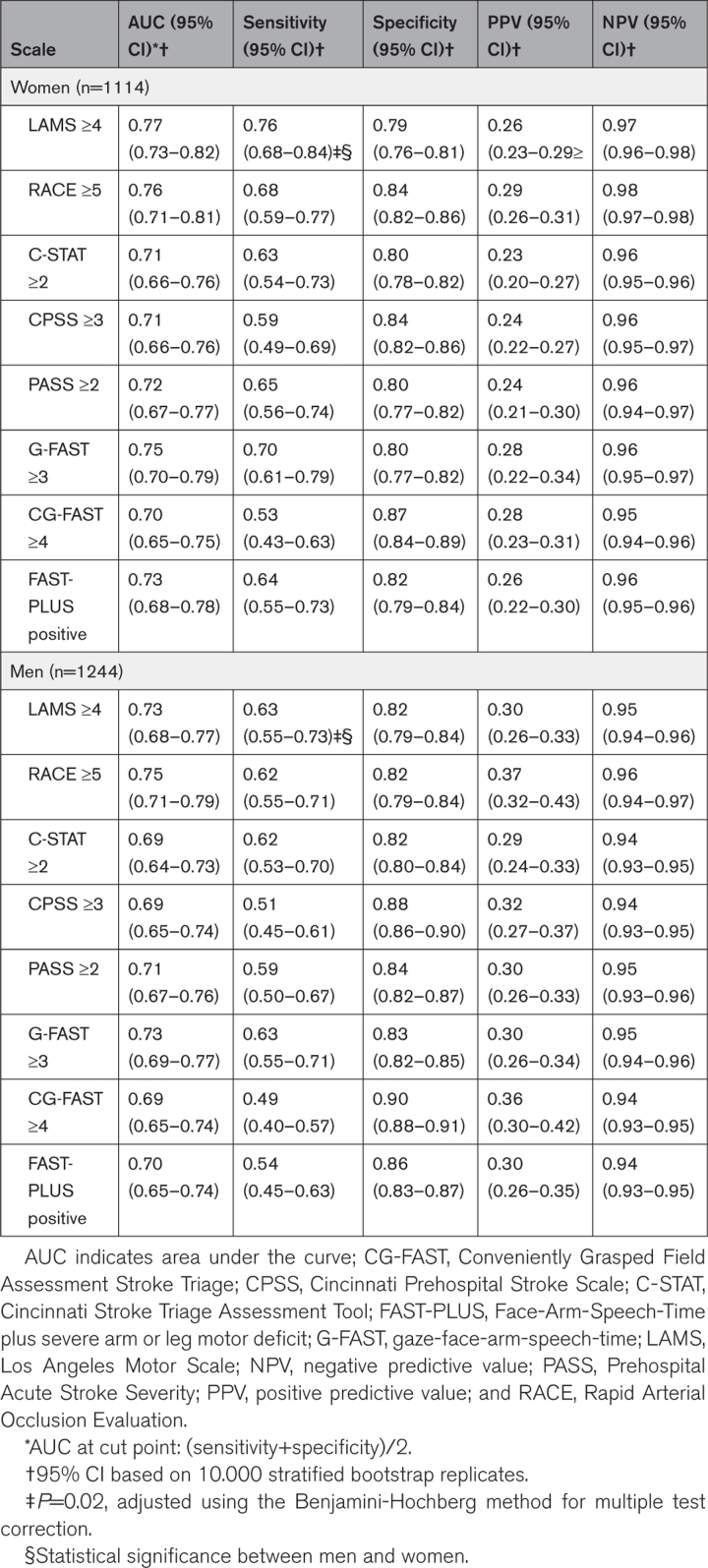
Diagnostic Performance of the Scales According to Prespecified Cut Points Stratified by Sex

The only statistically significant difference between sexes was the sensitivity of the Los Angeles Motor Scale, which was substantially higher in women than in men (0.76 versus 0.63; *P*=0.02). Results were largely similar following stratification for cohort (Tables S4 and S5) and within the subgroup of patients with confirmed stroke (Table S6).

## DISCUSSION

In this pooled analysis with individual patient data of 2 prospective cohort studies, we found similar diagnostic performance of 8 commonly used aLVO scales with generally high detection rates of aLVO in both men and women. The RACE scale had the highest PPV in both sexes along with the highest negative predictive value as well.

We found only minor sex differences in the performance of aLVO scales. One potential rationale for this observation is that these scales typically encompass focal cortical deficits (such as head or gaze deviation, aphasia, or neglect), which our prior research revealed to be comparable between men and women in the context of stroke presentation. Furthermore, it is worth noting that the majority of aLVO scales do not encompass focal deficits of the posterior circulation (like visual disturbances) or nonfocal and subcortical neurological deficits, both of which our earlier investigation showed to diverge between men and women during stroke presentation. Only the Conveniently Grasped Field Assessment Stroke Triage scale contained the nonfocal item level of consciousness, but no other nonfocal neurological deficits were included in other aLVO scales. We also found that the RACE scale outperforms all other scales, which might be related to the composition of this scale. Different aspects of RACE such as the inclusion of cortical deficits (ie, aphasia, neglect, and gaze palsy) in combination with arm and leg motor deficits, which are sensitive indicators for LVO, and the large number of items to be assessed on a wide range scale may have maximized its ability to detect aLVO compared with other scales.^22^

In a secondary analysis of the RACECAT trial (Transfer to the Closest Local Stroke Center vs Direct Transfer to Endovascular Stroke Center of Acute Stroke Patients With Suspected Large Vessel Occlusion in the Catalan Territory) in Catalonia, Spain, investigating prehospital transfer protocols in patients with spontaneous ICH, it was found that among 302 included patients, direct transfer to an EVT-capable center resulted in worse functional outcomes at 90 days compared with local transfer.^23^ Bypassing the closest stroke center reduced chances of functional independence at 90 days for patients with ICH. Delays in early blood pressure control and anticoagulation reversal at the closest center may have contributed to hematoma expansion and worsened outcomes.

The transferability of the RACECAT trial results from Catalonia, Spain, to the Netherlands is limited due to variations in the geographic distribution and proximity of local stroke centers to EVT-capable centers. The nonurban setting in Catalonia, where most local stroke centers were over 30 minutes away from EVT-capable centers, contrasts with prehospital LVO stroke triage protocols. These protocols usually advise bypassing non–EVT-capable centers only when transport to the closest EVT-capable stroke center would take <30 minutes. The observed outcomes in the RACECAT trial may be influenced by the nonurban characteristics and extended distances between local stroke centers and EVT-capable centers in the study area, aspects that do not align with situations in more urban or differently structured health care systems, such as those in the Netherlands.

Contrary to our expectation, we consistently observed a somewhat higher sensitivity and slightly lower specificity of aLVO scales in women. The lower specificity may be due to the higher frequency of stroke mimics in women with suspected stroke. Another factor contributing to more positive test results is the frequency of hemiparesis. Notably, in the group of patients with suspected stroke as assessed by ambulance paramedics, the final diagnosis was indeed a stroke in 64% of women and 74% of men. Early identification and triage of patients with LVO are crucial for swift access to specialized stroke centers, improving the chances of successful interventions and overall outcomes. However, the higher rate of stroke mimics in women may lead to suboptimal resource utilization, potentially causing treatment delays for those with actual strokes. Addressing this discrepancy necessitates targeted educational initiatives for ambulance paramedics to improve their ability to differentiate between stroke and mimics, ultimately contributing to improved prehospital triage.

While numerous studies on performance of aLVO scales in a population of patients with suspected stroke have been published, we found only 1 publication, which reported on sex differences in prehospital triage of patients with suspected stroke.^24^ This Swedish prospective observational cohort study included 2905 patients and investigated the Stockholm Stroke Triage System in which assessment of hemiparesis is combined with teleconsultation. No significant sex differences in performance metrics were observed. There were substantial differences in performance between the Stockholm Stroke Triage System in the Swedish study and the aLVO scales evaluated in our study. However, because the Stockholm Stroke Triage System was a teleconsultation-based triage system, direct comparison between this system and the scales evaluated in our study is not possible.

This study has several limitations. First, we only included suspected patients with stroke who presented within 6 hours after symptom onset because of the availability of computed tomography angiography, which was not routinely performed outside the 6-hour time window, resulting in 962 patients being excluded. This time restriction limits generalizability for patients presenting outside 6 hours after onset. However, we do not have reasons to assume that after this arbitrary time frame scale performance related to sex will be different than our findings. Second, we lack information on patients not identified by ambulance paramedics on the scene, as our data are limited to those with a suspicion of acute stroke as assessed by ambulance paramedics. Patients not meeting these criteria, whether arriving at the hospital for a different reason or independently at the emergency department without ambulance involvement, were not included in our studies. Third, aLVO scale scores were missing for 5% to 15% of the patients, and there were no substantial sex differences in missing aLVO scale scores. Fourth, 2 scales (the Gaze, Facial Asymmetry, Level of Consciousness, Extinction/Inattention scale, and the Field Assessment Stroke Triage for Emergency Destination scale) could not be investigated in the pooled database, because these could not be reconstructed from the PRESTO data.^25,26^ Fifth, information on gender was not recorded in our study. Consequently, our results cannot be extrapolated to gender-diverse populations.

The strengths of our study are our large real-world population including all patients with suspected stroke triaged in the field by ambulance paramedics. Therefore, our study provides a fair clinical evaluation of diagnostic performance of scales as used in prehospital clinical practice. In addition, our large sample size allowed us to study diagnostic performance of scales with sufficient precision. Last, collection of data was conducted in a prospective manner, which limited the amount of missing data.

## CONCLUSIONS

Our results indicate that the 8 currently most used aLVO triage scales show similar diagnostic performance in men and women. Implementation of RACE may help optimize prehospital identification of aLVO in men as well as in women with suspected stroke.

## ARTICLE INFORMATION

### Sources of Funding

LPSS (Leiden Prehospital Stroke Study) was funded by the Dutch Brain Foundation (grant, HA20 15.01.02), the Dutch Innovation Fund (grant, 3.240), and Health-Holland (grant, LSHM16041). The PRESTO (Prehospital Triage of Patients With Suspected Stroke Study) was funded by the BeterKeten collaboration and Theia Foundation (Zilveren Kruis).

### Disclosures

Dr Wermer reports receiving Clinical Established Investigator grant 2016T086 from the Dutch Heart Foundation and VIDI grant 9171337 from the Netherlands Organization for Health Research and Development (ZonMw) during the conduct of the original LPSS (Leiden Prehospital Stroke Study). Dr Kruyt reports receiving grant HA20 15.01.02 from the Dutch Brain Foundation, grant 3.240 from the Dutch Innovation Funds, and grant LSHM16041 from Health-Holland during the conduct of the study. Dr Roozenbeek reports funding from the Dutch Heart Foundation and the Netherlands Organization for Health Research and Development (ZonMw) during the conduct of this study, paid to the institution. Dr Dippel reports funding from the Dutch Heart Foundation, Brain Foundation Netherlands, the Netherlands Organisation for Health Research and Development, Health-Holland Top Sector Life Sciences & Health, and unrestricted grants from Penumbra Inc, Stryker, Medtronic, Thrombolytic Science, LLC, and Cerenovus for research, all paid to institution outside the submitted work. Dr van den Wijngaard reports compensation from Philips for consultant services, compensation from Medtronic for consultant services, and stock holdings in Neurophyxia BV. The other authors report no conflicts.

### Supplemental Material

STROBE checklist

Tables S1–S6

## APPENDIX

LPSS/PRESTO collaborators: Leo A.M. Aerden, Kees C.L. Alblas, Jeannette Bakker, Eduard van Belle, Timo Bevelander, Jan Bosch, Bianca Buijck, Jasper D. Daems, Luuk Dekker, Diederik W.J. Dippel, Tamara Dofferhoff-Vermeulen, Pieter Jan van Doormaal, Kirsten R.I.S. Dorresteijn, Dion Duijndam, Martijne H.C. Duvekot, Roeland P.J. van Eijkelenburg, Adriaan C.G.M. van Es, Jan-Hein Hensen, Amber Hoek, Henk Kerkhoff, Loet M.H. Kloos, Gaia T. Koster, Nyika D. Kruyt, Jan Willem Kuiper, Karlijn F. de Laat, Arnoud M. de Leeuw, Hester F. Lingsma, Aad van der Lugt, Geert Lycklama À Nijeholt, Lisette Maasland, Bruno J.M. van Moll, Walid Moudrous, Laus J.M.M. Mulder, T. Truc My Nguyen, Anja Noordam-Reijm, Erick Oskam, Aarnout Plaisier, Bob Roozenbeek, Anouk D. Rozeman, Els L.L.M. de Schryver, Esmee Venema, Marieke J.H. Wermer, Ruben M. van de Wijdeven, Ido R. van den Wijngaard, Annemarie D. Wijnhoud, Merel L. Willeboer, Mirjam Woudenberg, Mandy M.A. van der Zon, Erik W. van Zwet, Egon D. Zwets, Stas A. Zylicz.

## Supplementary Material
