# Supplementary material for: Sex Differences in Prehospital Identification of Large Vessel Occlusion in Patients With Suspected Stroke
Source: Stroke. 2024 Feb 1;55(3):548–54. doi: 10.1161/STROKEAHA.123.044898 (PMC10896195; doi:10.1161/STROKEAHA.123.044898)
Supplement: Supplementary file 1 [file str-55-548-s001.pdf]

SUPPLEMENTAL MATERIAL

Table S1. Overview of included clinical items in each scale

	CG-FAST ≥4	CPSS ≥3	C-STAT ≥2	FAST-PLUS positive	G-FAST ≥3	LAMS ≥4	RACE ≥5	PASS ≥2
Facial droop	✓	✓	-	✓	✓	✓	✓	-
Motor arm	✓	✓	✓	✓	✓	✓	✓	✓
Motor leg	-	-	-	✓	-	-	✓	-
Speech disturbance (aphasia or dysarthria)	✓	✓	-	✓	✓	-	✓	-
Gaze deviation	✓	-	✓	-	✓	-	✓	✓
Agnosia	-	-	-	-	-	-	✓	-
Grip strength	-	-	-	-	-	✓	-	-
Questions	✓	-	✓	-	-	-	-	✓
Commands	-	-	✓	-	-	-	✓	-

Table S2. Percentage negative, positive and missing scores per prehospital aLVO scale

Scale	Negative	Positive	Missing
LAMS	1679 (71.2)	474 (20.1)	205 (8.7)
RACE	1740 (73.8)	351 (14.9)	267 (11.3)
C-STAT	1771 (75.1)	463 (19.6)	124 (5.3)
CPSS	1870 (79.3)	355 (15.1)	133 (5.6)
PASS	1747 (74.1)	473 (20.1)	138 (5.9)
G-FAST	1727 (73.2)	484 (20.5)	147 (6.2)
CG-FAST	1923 (81.6)	309 (13.1)	126 (5.3)
FAST-PLUS	1596 (67.7)	400 (17.0)	362 (15.4)

Data are n (%). Abbreviations: CG-FAST, Conveniently-Grasped Field Assessment Stroke Triage; CPSS, Cincinnati Prehospital Stroke Scale; C-STAT, Cincinnati Stroke Triage Assessment Tool; FAST-PLUS, Face-Arm-Speech-Time plus severe arm or leg motor deficit; G-FAST, gaze-face-arm-speech-time; LAMS, Los Angeles Motor Scale; NPV, negative predictive value; PASS, Prehospital Acute Stroke Severity; PPV, positive predictive value; RACE, Rapid Arterial Occlusion Evaluation.

Table S3. Percentage missing scores per prehospital aLVO scale stratified by sex

Scale	Women with missing aLVO scores (n=226)	Men with missing aLVO scores (n=196)
LAMS	105 (9.4)	100 (8.0)
RACE	139 (12.5)	128 (10.3)
C-STAT	60 (5.4)	64 (5.1)
CPSS	65 (5.8)	68 (5.5)
PASS	65 (5.8)	73 (5.9)
G-FAST	68 (6.1)	79 (6.4)
CG-FAST	57 (5.1)	69 (5.5)
FAST-PLUS	192 (17.2)	170 (13.7)

Data are n (%). Abbreviations: CG-FAST, Conveniently-Grasped Field Assessment Stroke Triage; CPSS, Cincinnati Prehospital Stroke Scale; C-STAT, Cincinnati Stroke Triage Assessment Tool; FAST-PLUS, Face-Arm-Speech-Time plus severe arm or leg motor deficit; G-FAST, gaze-face-arm-speech-time; LAMS, Los Angeles Motor Scale; NPV, negative predictive value; PASS, Prehospital Acute Stroke Severity; PPV, positive predictive value; RACE, Rapid Arterial Occlusion Evaluation.

Table S4. Diagnostic performance of the scales according to prespecified cut points stratified by sex in the LPSS study

Prediction scale	AUC (95% CI)*†	Sensitivity (95% CI)†	Specificity (95% CI)†	PPV (95% CI)†	NPV (95% CI)†
**Women (n=635)**					
LAMS ≥ 4	0.82 (0.74-0.89)	0.77 (0.61-0.90)	0.86 (0.83-0.89)	0.25 (0.20-0.31)	0.98 (0.97-0.99)
RACE ≥ 5	0.83 (0.76-0.90)	0.79 (0.64-0.91)	0.87 (0.84-0.90)	0.30 (0.24-0.37)	0.98 (0.97-0.99)
C-STAT ≥ 2	0.75 (0.68-0.82)	0.72 (0.56-0.85)	0.78 (0.75-0.82)	0.19 (0.15-0.23)	0.97 (0.96-0.99)
CPSS ≥ 3	0.74 (0.66-0.82)	0.60 (0.43-0.77)	0.88 (0.85-0.91)	0.25 (0.18-0.32)	0.97 (0.96-0.98)
PASS ≥ 2	0.76 (0.69-0.83)	0.72 (0.59-0.85)	0.80 (0.77-0.83)	0.21 (0.17-0.25)	0.97 (0.96-0.99)
G-FAST ≥ 3	0.79 (0.72-0.86)	0.76 (0.63-0.87)	0.81 (0.78-0.85)	0.23 (0.18-0.27)	0.98 (0.97-0.99)
CG-FAST ≥ 4	0.77 (0.69-0.85)	0.66 (0.50-0.79)	0.88 (0.86-0.91)	0.28 (0.22-0.35)	0.97 (0.96-0.98)
FAST-PLUS positive	0.80 (0.73-0.88)	0.77 (0.61-0.90)	0.83 (0.80-0.87)	0.26 (0.21-0.32)	0.98 (0.97-0.99)
**Men (n=684)**					
LAMS ≥ 4	0.69 (0.62-0.76)	0.49 (0.35-0.63)	0.89 (0.86-0.91)	0.29 (0.22-0.37)	0.95 (0.94-0.96)
RACE ≥ 5	0.71 (0.64-0.78)	0.49 (0.35-0.63)	0.92 (0.90-0.95)	0.39 (0.30-0.49)	0.95 (0.93-0.96)
C-STAT ≥ 2	0.71 (0.64-0.77)	0.56 (0.44-0.68)	0.85 (0.82-0.88)	0.30 (0.24-0.36)	0.95 (0.93-0.96)
CPSS ≥ 3	0.65 (0.59-0.72)	0.39 (0.26-0.51)	0.92 (0.90-0.94)	0.34 (0.25-0.44)	0.94 (0.92-0.95)
PASS ≥ 2	0.70 (0.63-0.76)	0.53 (0.41-0.66)	0.86 (0.83-0.89)	0.28 (0.22-0.35)	0.95 (0.93-0.96)
G-FAST ≥ 3	0.72 (0.65-0.78)	0.57 (0.45-0.69)	0.86 (0.83-0.89)	0.31 (0.24-0.37)	0.95 (0.94-0.96)
CG-FAST ≥ 4	0.65 (0.59-0.72)	0.38 (0.26-0.50)	0.93 (0.91-0.95)	0.36 (0.26-0.47)	0.94 (0.92-0.95)
FAST-PLUS positive	0.66 (0.59-0.74)	0.46 (0.32-0.60)	0.87 (0.84-0.90)	0.27 (0.20-0.35)	0.94 (0.92-0.95)

Abbreviations: CG-FAST, Conveniently-Grasped Field Assessment Stroke Triage; CPSS, Cincinnati Prehospital Stroke Scale; C-STAT, Cincinnati Stroke Triage Assessment Tool; FAST-PLUS, Face-Arm-Speech-Time plus severe arm or leg motor deficit; G-FAST, gaze-face-arm-speech-time; LAMS, Los Angeles Motor Scale; NPV, negative predictive value; PASS, Prehospital Acute Stroke Severity; PPV, positive predictive value; RACE, Rapid Arterial Occlusion Evaluation.

*AUC at cut point: (sensitivity + specificity)/2.

†95% CI based on 10,000 stratified bootstrap replicates.

Table S5. Diagnostic performance of the scales according to prespecified cut points stratified by sex in the PRESTO study

Scale	AUC (95% CI)*†	Sensitivity (95% CI)†	Specificity (95% CI)†	PPV (95% CI)†	NPV (95% CI)†
**Women (n=479)**					
LAMS ≥ 4	0.74 (0.68-0.80)	0.72 (0.60-0.84)	0.76 (0.71-0.80)	0.29 (0.24-0.34)	0.95 (0.93-0.97)
RACE ≥ 5	0.75 (0.69-0.81)	0.64 (0.52-0.76)	0.86 (0.83-0.89)	0.39 (0.32-0.46)	0.95 (0.93-0.96)
C-STAT ≥ 2	0.70 (0.64-0.77)	0.57 (0.45-0.69)	0.84 (0.80-0.87)	0.33 (0.26-0.40)	0.93 (0.92-0.95)
CPSS ≥ 3	0.70 (0.63-0.77)	0.55 (0.43-0.67)	0.85 (0.81-0.88)	0.33 (0.26-0.41)	0.93 (0.92-0.95)
PASS ≥ 2	0.71 (0.64-0.78)	0.60 (0.47-0.72)	0.82 (0.78-0.86)	0.31 (0.25-0.38)	0.94 (0.92-0.96)
G-FAST ≥ 3	0.73 (0.67-0.80)	0.66 (0.53-0.78)	0.81 (0.77-0.85)	0.32 (0.27-0.38)	0.94 (0.93-0.96)
CG-FAST ≥ 4	0.67 (0.59-0.75)	0.47 (0.34-0.60)	0.88 (0.85-0.91)	0.35 (0.27-0.44)	0.92 (0.91-0.94)
FAST-PLUS positive	0.69 (0.61-0.74)	0.57 (0.45-0.69)	0.82 (0.78-0.86)	0.30 (0.24-0.37)	0.93 (0.91-0.95)
**Men (n=560)**					
LAMS ≥ 4	0.74 (0.68-0.80)	0.69 (0.58-0.81)	0.79 (0.75-0.82)	0.29 (0.24-0.34)	0.95 (0.94-0.97)
RACE ≥ 5	0.78 (0.72-0.84)	0.69 (0.58-0.81)	0.87 (0.84-0.90)	0.41 (0.34-0.48)	0.96 (0.94-0.97)
C-STAT ≥ 2	0.64 (0.58-0.71)	0.44 (0.31-0.56)	0.85 (0.82-0.88)	0.27 (0.20-0.35)	0.92 (0.91-0.94)
CPSS ≥ 3	0.72 (0.65-0.78)	0.58 (0.45-0.69)	0.85 (0.82-0.88)	0.33 (0.27-0.40)	0.94 (0.93-0.96)
PASS ≥ 2	0.71 (0.64-0.77)	0.58 (0.45-0.69)	0.84 (0.80-0.87)	0.31 (0.25-0.37)	0.94 (0.92-0.96)
G-FAST ≥ 3	0.75 (0.69-0.81)	0.68 (0.56-0.79)	0.82 (0.79-0.86)	0.32 (0.27-0.38)	0.95 (0.94-0.97)
CG-FAST ≥ 4	0.72 (0.65-0.78)	0.53 (0.40-0.66)	0.90 (0.88-0.93)	0.40 (0.32-0.49)	0.94 (0.92-0.95)
FAST-PLUS positive	0.74 (0.68-0.80)	0.63 (0.50-0.74)	0.85 (0.82-0.88)	0.34 (0.28-0.41)	0.95 (0.93-0.96)

Abbreviations: CG-FAST, Conveniently-Grasped Field Assessment Stroke Triage; CPSS, Cincinnati Prehospital Stroke Scale; C-STAT, Cincinnati Stroke Triage Assessment Tool; FAST-PLUS, Face-Arm-Speech-Time plus severe arm or leg motor deficit; G-FAST, gaze-face-arm-speech-time; LAMS, Los Angeles Motor Scale; NPV, negative predictive value; PASS, Prehospital Acute Stroke Severity; PPV, positive predictive value; RACE, Rapid Arterial Occlusion Evaluation.

*AUC at cut point: (sensitivity + specificity)/2.

†95% CI based on 10,000 stratified bootstrap replicates.

Table S6. Diagnostic performance of the scales according to prespecified cut points stratified by sex in patients with confirmed stroke**

Prediction scale	AUC (95% CI)*†	Sensitivity (95% CI)†	Specificity (95% CI)†	PPV (95% CI)†	NPV (95% CI)†
**Women (n=716)**					
LAMS ≥ 4	0.78 (0.74-0.81)	0.76 (0.68-0.84)	0.78 (0.75-0.80)	0.27 (0.24-0.30)	0.98 (0.97-0.99)
RACE ≥ 5	0.77 (0.72-0.82)	0.68 (0.59-0.77)	0.85 (0.83-0.87)	0.28 (0.25-0.30)	0.98 (0.97-0.99)
C-STAT ≥ 2	0.72 (0.67-0.77)	0.63 (0.54-0.73)	0.81 (0.78-0.83)	0.24 (0.21-0.26)	0.97 (0.96-0.98)
CPSS ≥ 3	0.72 (0.67-0.77)	0.59 (0.49-0.69)	0.85 (0.83-0.87)	0.25 (0.23-0.28)	0.97 (0.96-0.98)
PASS ≥ 2	0.73 (0.68-0.78)	0.65 (0.56-0.74)	0.79 (0.76-0.81)	0.25 (0.22-0.29)	0.97 (0.95-0.98)
G-FAST ≥ 3	0.76 (0.71-0.80)	0.70 (0.61-0.79)	0.81 (0.78-0.83)	0.28 (0.21-0.33)	0.97 (0.96-0.98)
CG-FAST ≥ 4	0.71 (0.66-0.76)	0.53 (0.43-0.63)	0.86 (0.83-0.88)	0.27 (0.22-0.30)	0.96 (0.95-0.97)
FAST-PLUS positive	0.74 (0.69-0.79)	0.64 (0.55-0.73)	0.83 (0.80-0.85)	0.25 (0.21-0.29)	0.97 (0.96-0.97)
**Men (n=920)**					
LAMS ≥ 4	0.72 (0.67-0.76)	0.63 (0.55-0.73)	0.83 (0.80-0.85)	0.31 (0.27-0.34)	0.96 (0.95-0.97)
RACE ≥ 5	0.74 (0.70-0.78)	0.62 (0.55-0.71)	0.82 (0.79-0.84)	0.36 (0.31-0.42)	0.96 (0.95-0.97)
C-STAT ≥ 2	0.68 (0.63-0.72)	0.62 (0.53-0.70)	0.81 (0.78-0.83)	0.28 (0.23-0.32)	0.95 (0.94-0.96)
CPSS ≥ 3	0.68 (0.64-0.69)	0.51 (0.44-0.61)	0.89 (0.87-0.91)	0.33 (0.28-0.38)	0.95 (0.94-0.96)
PASS ≥ 2	0.70 (0.66-0.75)	0.59 (0.50-0.67)	0.83 (0.81-0.86)	0.31 (0.27-0.35)	0.96 (0.94-0.97)
G-FAST ≥ 3	0.72 (0.68-0.76)	0.63 (0.55-0.71)	0.84 (0.82-0.86)	0.31 (0.27-0.35)	0.96 (0.95-0.97)
CG-FAST ≥ 4	0.68 (0.64-0.70)	0.49 (0.40-0.57)	0.91 (0.89-0.92)	0.37 (0.31-0.43)	0.95 (0.94-0.96)
FAST-PLUS positive	0.69 (0.64-0.73)	0.54 (0.45-0.63)	0.87 (0.84-0.88)	0.31 (0.27-0.36)	0.95 (0.94-0.96)

Abbreviations: CG-FAST, Conveniently-Grasped Field Assessment Stroke Triage; CPSS, Cincinnati Prehospital Stroke Scale; C-STAT, Cincinnati Stroke Triage Assessment Tool; FAST-PLUS, Face-Arm-Speech-Time plus severe arm or leg motor deficit; G-FAST, gaze-face-arm-speech-time; LAMS, Los Angeles Motor Scale; NPV, negative predictive value; PASS, Prehospital Acute Stroke Severity; PPV, positive predictive value; RACE, Rapid Arterial Occlusion Evaluation.

*AUC at cut point: (sensitivity + specificity)/2.

**Ischemic stroke, intracranial hemorrhage, and TIA.

†95% CI based on 10,000 stratified bootstrap replicates.
